# Sinus Bradycardia in Carriers of the *SCN5A*-1795insD Mutation: Unraveling the Mechanism through Computer Simulations

**DOI:** 10.3390/ijms19020634

**Published:** 2018-02-23

**Authors:** Ronald Wilders

**Affiliations:** Department of Medical Biology, Academic Medical Center, University of Amsterdam, 1105 AZ Amsterdam, The Netherlands; r.wilders@amc.uva.nl; Tel.: +31-20-56-65229

**Keywords:** heart, sinoatrial node, electrophysiology, long-QT syndrome, sinus bradycardia, sick sinus syndrome, genetics, sodium current, ion channels, computer simulations

## Abstract

The *SCN5A* gene encodes the pore-forming α-subunit of the ion channel that carries the cardiac fast sodium current (*I_Na_*). The 1795insD mutation in *SCN5A* causes sinus bradycardia, with a mean heart rate of 70 beats/min in mutation carriers vs. 77 beats/min in non-carriers from the same family (lowest heart rate 41 vs. 47 beats/min). To unravel the underlying mechanism, we incorporated the mutation-induced changes in *I_Na_* into a recently developed comprehensive computational model of a single human sinoatrial node cell (Fabbri–Severi model). The 1795insD mutation reduced the beating rate of the model cell from 74 to 69 beats/min (from 49 to 43 beats/min in the simulated presence of 20 nmol/L acetylcholine). The mutation-induced persistent *I_Na_* per se resulted in a substantial increase in beating rate. This gain-of-function effect was almost completely counteracted by the loss-of-function effect of the reduction in *I_Na_* conductance. The further loss-of-function effect of the shifts in steady-state activation and inactivation resulted in an overall loss-of-function effect of the 1795insD mutation. We conclude that the experimentally identified mutation-induced changes in *I_Na_* can explain the clinically observed sinus bradycardia. Furthermore, we conclude that the Fabbri–Severi model may prove a useful tool in understanding cardiac pacemaker activity in humans.

## 1. Introduction

The ‘fast sodium current’ (*I_Na_*), which flows through Na_V_1.5 sodium channels, is a key player in the electrical activity of the human heart. The cardiac-specific Na_V_1.5 protein, encoded by the *SCN5A* gene, is the pore-forming α-subunit of the channel. The large and fast influx of sodium ions through the Na_V_1.5 channels is not only responsible for the fast upstroke of individual atrial and ventricular cardiomyocytes, but also for the fast impulse propagation in the atrial and ventricular tissue. Thus, *I_Na_* is an important determinant of the PQ interval, or PR interval, and the QRS duration on the body surface electrocardiogram (ECG) with the letters P, Q, R, S, and T assigned to its deflections [1,2,3,4].

Mutations in genes encoding ion channel-related proteins may result in inherited arrhythmia disorders [5,6], in particular the long QT syndrome (LQTS), which shows an estimated prevalence of 1:2000 [7] and is the most commonly encountered inherited arrhythmia disorder in clinical practice (≈35% [8]). LQTS type 3 (LQT3) is caused by gain-of-function mutations in the *SCN5A* gene. Slowed or incomplete inactivation of the Na_V_1.5 channel results in an additional inward current, known as the late or persistent sodium current (*I_pst_*), during the plateau phase of the ventricular action potential. This additional inward current leads to an increase in the ventricular action potential duration and thereby to a prolongation of the QT interval on the ECG [9,10,11,12,13,14,15]. The estimated prevalence of LQT3 among LQTS patients is ≈10% [16,17].

An intriguing and widely studied mutation among the mutations in *SCN5A* associated with LQT3 is 1795insD, which is characterized by the insertion of 3 nucleotides (TGA) at position 5537 in the DNA sequence of the *SCN5A* gene, causing an insertion of aspartic acid (1795insD) in the C-terminal domain of the Na_V_1.5 protein [18]. Carriers of this mutation may not only present with LQT3, but also with ECG features of sinus bradycardia, progressive cardiac conduction disease, and Brugada syndrome, thus creating the first described arrhythmic ‘overlap syndrome’ [19,20]. Interestingly, 1795insD is supposed to be a gain-of-function mutation in light of the QT prolongation, but a loss-of-function mutation in light of the sinus bradycardia, progressive cardiac conduction disease, and Brugada syndrome [18].

Acquisition of clinical data on carriers of the 1795insD mutation, which is a founder mutation in the Netherlands [20], started in 1958 with the presentation of abnormalities on a routine ECG [21]. Fifty years later, clinical data were available on 378 family members of whom 149 carried the 1795insD mutation in the *SCN5A* gene [20]. Holter recording in 1795insD patients and non-affected family members (“non-carriers”) has revealed sinus bradycardia with an ≈11% decrease in minimum, average, and maximum heart rate [21], as detailed in Table 1. The bradycardic effect is not as large as for some other mutations in *SCN5A*, e.g., ΔKPQ [22], or in *HCN4*, another gene associated with “sick sinus syndrome” [23]. Yet, it may be severe. This is because carriers of the 1795insD mutation, like carriers of other LQT3 mutations [24], show an excessive pro-arrhythmic QT prolongation at low heart rates [25], which may favour the development of early afterdepolarizations and torsades de pointes arrhythmias at rest or during sleep [24]. Accordingly, carriers of the mutation are prophylactically treated with an anti-bradycardia pacemaker as a preventive measure against the premature (mostly nocturnal) sudden cardiac deaths that are associated with the mutation [26].

The 1795insD mutation was identified in 1998 [21]. It was first described in 1999 by Bezzina et al. [18], who also presented the first in vitro data on the functional effects of the mutation. They had carried out voltage clamp experiments on wild-type and 1795insD mutant Na^+^ channels expressed in *Xenopus* oocytes. In line with the clinically observed conduction delays and Brugada phenotype, mutational loss-of-function effects had been found. The *I_Na_* steady-state activation curve was shifted by +8.1 mV, whereas the steady-state inactivation curve was shifted by −7.3 mV. Also, the maximal *I_Na_* amplitude, as determined by voltage clamp steps from a holding potential of −100 mV, was reduced by 78%. In subsequent studies by Veldkamp et al. [27,28], who carried out patch clamp experiments on HEK-293 cells transfected with wild-type or 1795insD mutant cDNA, a mutational gain-of-function effect was also observed: the mutant channels showed a persistent current of 1.4 ± 0.2% (mean ± standard error of the mean (SEM); percent of *I_Na_* peak current amplitude) at −20 mV [27] and this *I_pst_* ranged from 0.8 ± 0.2% to 1.9 ± 0.8% at voltages from −50 to 0 mV (with a value of 1.6 ± 0.5% at −20 mV) [28]. In the initial study [27], the wild-type channels also showed a persistent current (of 0.4 ± 0.1% at −20 mV), but in the subsequent study [28] this wild-type *I_pst_* was not only effectively zero at −40 mV (with a reported value of 0.04 ± 0.14%), but also at other membrane potential values, including −20 mV.

Because human sinoatrial (SA) nodal tissue is not widely available for in vitro experiments, data on the functional role of Na_V_1.5 channels in the pacemaker activity of the human SA node are scarce. However, there is substantial evidence, albeit more or less indirect, for such a role. First, several mutations in *SCN5A* are associated with sinus bradycardia [29,30]. Second, human heart rate is affected by toxins and drugs that have an effect on the conductance or kinetics of Na_V_1.5 channels [28]. Third, Chandler et al. [31] carried out an extensive study on the molecular architecture of the human SA node and demonstrated the expression of Na_V_1.5 mRNA and protein in the nodal tissue. Fourth, Verkerk et al. [32] observed large inward currents with characteristics of *I_Na_* in two out of three cells in their unique patch clamp experiments on isolated human SA nodal pacemaker cells.

Recently, Fabbri et al. [33] presented the first comprehensive mathematical model of the spontaneous electrical activity of a human SA nodal cell. This Fabbri–Severi model is strictly based on and constrained by the available electrophysiological data. Thus, it also includes equations for *I_Na_*. The Fabbri–Severi model enabled us to carry out detailed computer simulations to assess each of the loss-of-function and gain-of-function effects of the 1795insD mutation (i.e., the reduction in *I_Na_* conductance, the shifts in the steady-state activation and inactivation curves, and the persistent current). Our simulation results provide insight into the loss-of-function and gain-of-function effects of the 1795insD mutation as well as the complex interaction of these effects underlying the sinus bradycardia.

## 2. Results

### 2.1. Implementation of the 1795insD Mutation

**Figure 1:**
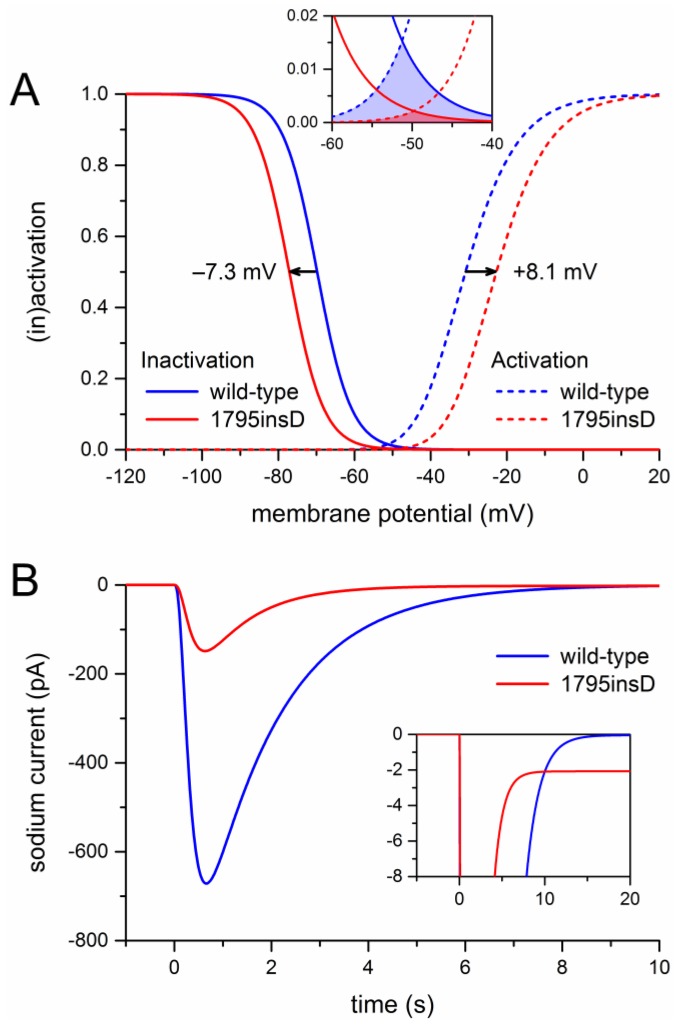
Incorporation of the effects of the 1795insD mutation observed in expression systems into the Fabbri–Severi model of a human SA nodal pacemaker cell. (**A**) Mutation-induced shifts of +8.1 and −7.3 mV, as indicated by horizontal arrows, in the steady-state *I_Na_* activation and inactivation curves of the Fabbri–Severi model cell. The inset shows the effects of the shifts on the *I_Na_* window current. (**B**) Current traces of the wild-type and the 1795insD mutant *I_Na_* upon a simulated voltage clamp step from a holding potential of −100 mV to a test potential of −25 mV. The inset shows the persistent component of the mutant *I_Na_*.

Before we could start to carry out the desired computer simulations, we had to determine the appropriate parameter settings of the sodium current of the Fabbri–Severi model cell. First, we split the *I_Na_* of the Fabbri–Severi model cell into a wild-type and a mutant component, thus representing the heterozygous nature of the mutation, as detailed in Section 4.1 and Section 4.2. For the wild-type component we used the regular *I_Na_* equations of the model with the fully-activated conductance (*g_Na_*) set to 50% of the control value. To implement the experimentally observed +8.1 and −7.3 mV shifts in the steady-state activation and inactivation curves of the 1795insD mutant *I_Na_* [18], we applied +8.1 and −7.3 mV shifts to the activation and inactivation equations of the mutant component. The resulting steady-state activation and inactivation curves of the wild-type and mutant components are shown in Figure 1A. It is important to note that the shifts result in a large reduction in “window current” (Figure 1A, inset).

Next, we simulated a voltage clamp step from a holding potential of −100 mV to a test potential of −25 mV. Using this protocol, Bezzina et al. [18] had found that the peak mutant current amounted to 22% of the peak wild-type current. It turned out that we had to set the mutant *g_Na_* to 50% of the corresponding wild-type value to obtain the experimentally observed 78% reduction in peak current. The resulting current traces are shown in Figure 1B. The blue trace is obtained with the control sodium current of the Fabbri–Severi model cell (100% wild-type), whereas the red trace is obtained with the mutant *I_Na_* equations (100% mutant), as in the experimental settings. The 78% reduction in peak current is not only due to the 50% reduction in *g_Na_*, but also to the +8.1 and −7.3 mV shifts in the steady-state activation and inactivation curves, which by themselves already create a reduction in peak current. At the same time, 0.6% of the mutant channels were made non-inactivating, as opposed to none of the wild-type channels, to incorporate a persistent current with an amplitude of 1.4% of the peak current (Figure 1B, inset), in line with the experimentally observed persistent current at −20 mV of 1.4 ± 0.2% (mean ± SEM, *n* = 7) [27] and of 1.6 ± 0.5% (mean ± SEM, *n* = 7) [28]. With the 0.6% fraction of non-inactivating channels, *I_pst_* ranged from 1.0 to 1.5% at voltages from −50 to 0 mV, in line with the aforementioned experimentally observed range from 0.8 ± 0.2% to 1.9 ± 0.8% (mean ± SEM, *n* = 6) [28].

Altogether, the parameter settings of the mutant component of the model *I_Na_* differed from those of the wild-type component in four respects, based on data from voltage clamp experiments [18,27,28]: a +8.1 mV shift in activation, a −7.3 mV shift in inactivation, a 50% reduction in *g_Na_*, and a 0.6% fraction of non-inactivating channels (underlying the experimentally observed persistent current). Computer simulations allowed us to determine how each of these parameters affected the pacemaker activity of the Fabbri–Severi model cell, thus gaining insight into the mechanisms underlying the clinically observed sinus bradycardia among carriers of the 1795insD mutation.

### 2.2. Effects of the Shifts in Steady-State Activation and Inactivation

We started our simulations with the default Fabbri–Severi model (normal autonomic tone). First, we assessed the effects of the shifts in activation and inactivation. Because the changes in membrane potential over the time course of a human SA nodal action potential, in particular during diastolic depolarization, are relatively slow, the amount of open, i.e., conducting, Na^+^ channels is largely determined by the region of overlap of the steady-state activation and inactivation curves, which defines the “window current”. As can be appreciated from Figure 1A (inset), either shift already results in a substantial decrease in window current. The decrease is even larger in the presence of both shifts.

**Figure 2:**
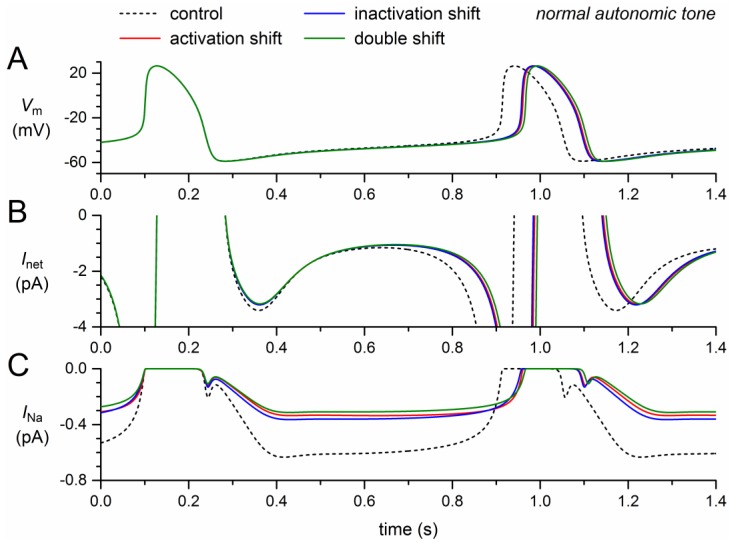
Effect of a +8.1 mV shift in steady-state activation and a −7.3 mV shift in steady-state inactivation as observed for the 1795insD mutation in *SCN5A* on the electrical activity of the Fabbri–Severi model cell at normal beating rate (normal autonomic tone). (**A**) Membrane potential (*V_m_*). (**B**) Net membrane current (*I_net_*). (**C**) Fast sodium current (*I_Na_*). Effect of the shift in steady-state activation per se (red traces), the shift in steady-state inactivation per se (blue traces), and the combined effect (green traces). Note the difference in ordinate scales between panels B and C. In the model, *I_Na_* is split into a wild-type and a mutant component, thus representing the effect of a heterozygous mutation.

If the steady-state activation curve is shifted by +8.1 mV, the model *I_Na_* decreases by almost 50% (Figure 2C, solid red trace versus dashed black trace), which implies that the remaining *I_Na_* is almost completely carried by the wild-type component of the model *I_Na_*. A slightly smaller decrease is observed if the steady-state inactivation curve is shifted by −7.3 mV (Figure 2C, blue trace). In either case, the “mutant *I_Na_*” is already largely inhibited, which explains why the further decrease in case of a combination of the two shifts is not very impressive (Figure 2C, green trace).

The decrease in *I_Na_* results in a small decrease in the net inward current during diastolic depolarization (Figure 2B). In the absence of a noticeable effect of *I_Na_* on the action potential, the resulting decrease in diastolic depolarization rate leads to an increase in cycle length (Figure 2A), which is prolonged by 46 ms (+5.7%) upon the +8.1 mV shift in steady-state activation and by 44 ms (+5.4%) upon the −7.3 mV shift in steady-state inactivation (Table 2). The prolonging effects are far from additive: the prolongation amounts to 53 ms (+6.5%) if the two shifts are combined.

### 2.3. Effects of the Reduction in Fully-Activated Conductance and the Incorporation of a Persistent Current

**Figure 3:**
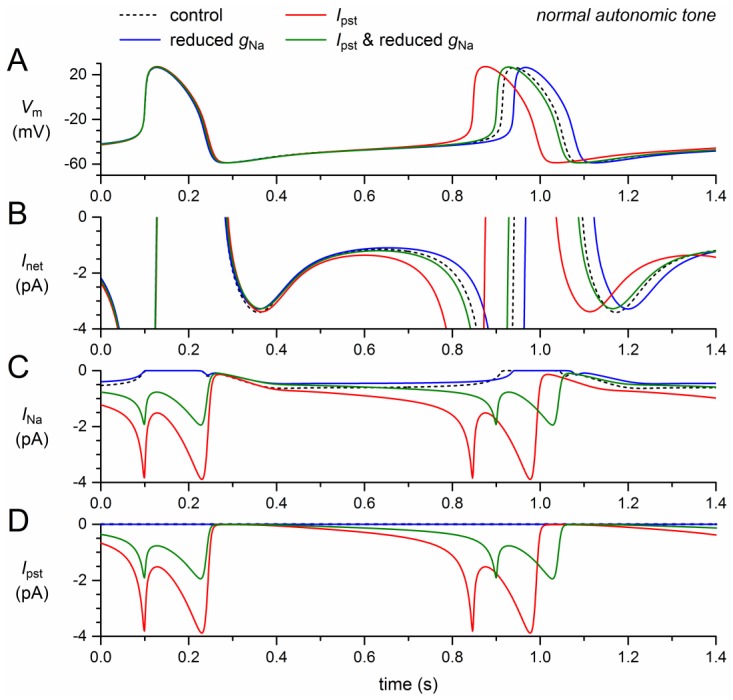
Effect of a 50% decrease in fully-activated conductance (*g_Na_*) and a 1.0–1.5% persistent current (*I_pst_*) as observed for the 1795insD mutation in *SCN5A* on the electrical activity of the Fabbri–Severi model cell at normal beating rate (normal autonomic tone). (**A**) Membrane potential (*V_m_*). (**B**) Net membrane current (*I_net_*). (**C**) Fast sodium current (*I_Na_*). (**D**) Persistent component of the fast sodium current (*I_pst_*). Effect of the reduced *g_Na_* per se (blue traces), the persistent *I_Na_* per se (red traces), and the combined effect (green traces). In the model, *I_Na_* is split into a wild-type and a mutant component, thus representing the effect of a heterozygous mutation.

Next, we assessed the effects of the reduction in fully-activated conductance and, on the other hand, the incorporation of a persistent current, *I_pst_*. Like the mutation-induced shifts in activation and inactivation, the 50% decrease in *g_Na_* exhibits a loss-of-function effect on the model *I_Na_*. The remaining model *I_Na_*, composed of wild-type and mutant *I_Na_*, thus amounts to ≈75% of its control (Figure 3C, solid blue trace versus dashed black trace). The resulting decrease in net inward current during diastolic depolarization (Figure 3B) leads to a 26-ms increase in cycle length (+3.2%; Figure 3A, Table 2).

The persistent current, on the other hand, has a gain-of-function effect. The sodium influx through the small amount of non-inactivating channels increases during diastole and reaches a peak of almost 4 pA during the upstroke of the action potential and its repolarization (Figure 3A,C, red traces), where the *I_Na_* driving force is larger than near the top of the action potential. During the systolic phase of the action potential, the non-persistent component of *I_Na_* is effectively zero, which explains the similarity of the *I_Na_* and *I_pst_* traces in Figure 3C,D. The additional inward current during the systolic phase leads to a slight increase in action potential duration, amounting to 6.5 ms at 90% repolarization (Figure 3A, Table 2). This effect by itself would prolong the cycle length. However, it is much smaller than the shortening effect of the additional inward current that now flows during diastolic depolarization (Figure 3A,C). The net effect is a shortening of the cycle length by 66 ms (−8.1%; Figure 3A, Table 2).

The gain-of-function effect of *I_pst_* is largely counteracted by the loss-of-function effect of the 50% decrease in *g_Na_* (Figure 3, green traces). The additional inward current is now limited to the second half of diastolic depolarization (Figure 3B,C) and the cycle length is shortened by only 12 ms (−1.5%; Figure 3A, Table 2).

### 2.4. Net Effect: Loss of Function Versus Gain of Function

The 1795insD mutation exhibits loss-of-function as well as gain-of-function effects. In the next set of simulations, we quantified the loss-of-function effects per se, the gain-of-function effect per se, and the combined effect.

**Figure 4:**
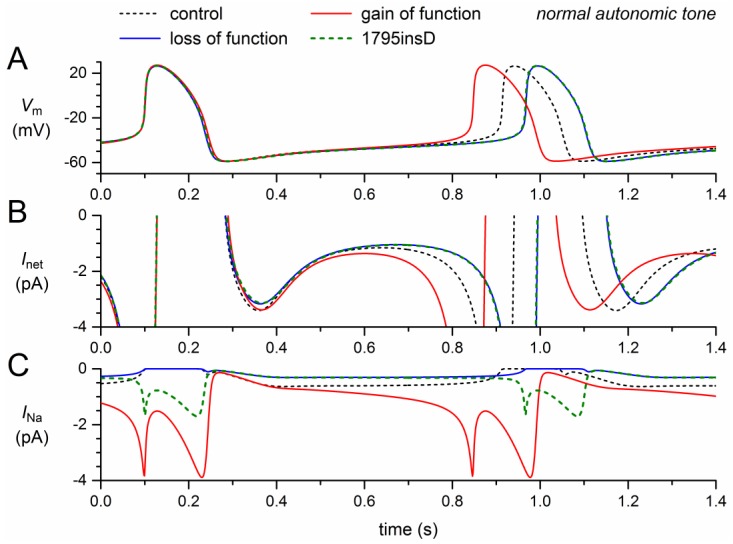
Changes in the electrical activity of the Fabbri–Severi model cell at normal beating rate (normal autonomic tone) due to loss-of-function (reduced *g_Na_* and shifts in the steady-state activation and inactivation curves) and gain-of-function (*I_pst_*) effects as observed for the 1795insD mutation in *SCN5A*. (**A**) Membrane potential (*V_m_*). (**B**) Net membrane current (*I_net_*). (**C**) Fast sodium current (*I_Na_*). Effect of the loss-of-function effects per se (blue traces), the gain-of-function effects per se (red traces), and the combined effect (dashed green traces). In the model, *I_Na_* is split into a wild-type and a mutant component, thus representing the effect of a heterozygous mutation.

Incorporation of the +8.1 mV shift in activation, the −7.3 mV shift in inactivation, and the 50% decrease in *g_Na_*, all loss-of-function effects, resulted in a dramatic decrease in model *I_Na_* (Figure 4C, solid blue trace versus dashed black trace). Effectively, the remaining *I_Na_* was carried by the wild-type channels. The resulting decrease in net inward current (Figure 4B) led to a 54-ms increase in cycle length (6.6%; Figure 4A, Table 2), which is only 0.8 ms more than the prolongation obtained with the shifts in activation and inactivation (6.5%; Section 2.2, Table 2).

We already noticed that *I_pst_* per se shortened the cycle length substantially, by 66 ms (Section 2.3, Table 2), raising the question of whether the loss-of-function effects of the 1795insD mutation could cancel its gain-of-function effect and even prolong the cycle length to a clear extent. The answer is shown in the dashed green traces of Figure 4, which are almost superimposed with the solid blue traces that show the loss-of-function effects per se. The net effect of the loss-of-function and gain-of-function effects appears to be a 53-ms increase (+6.5%) in cycle length (Figure 4A), which is almost identical to the 54-ms increase in cycle length in case of the loss-of-function effects per se (Table 2). This is because *I_pst_* is so strongly reduced by the combined loss-of-function effects (Figure 4C) that its shortening effect on the diastolic depolarization phase is almost cancelled. On the other hand, there is sufficient *I_pst_* left to increase the action potential duration at 90% repolarization by 2.0 ms (Figure 4A, Table 2), thus increasing the cycle length rather than decreasing it.

### 2.5. Net Effect at Low Heart Rate

The above simulations were all carried out with the default Fabbri–Severi model, i.e., under normal autonomic tone. In light of the 1795insD mutation, it is important to know whether the observed bradycardic effects of the mutation also occur at a low heart rate. We therefore repeated the simulations of Figure 4 during the simulated administration of 20 nmol/L acetylcholine (ACh), which reduces the beating rate of the model cell from 74 to 49 beats/min through its effect on several membrane ionic currents as well as the Ca^2+^ uptake by the sarcoplasmic reticulum, as detailed in Section 4.3.

**Figure 5:**
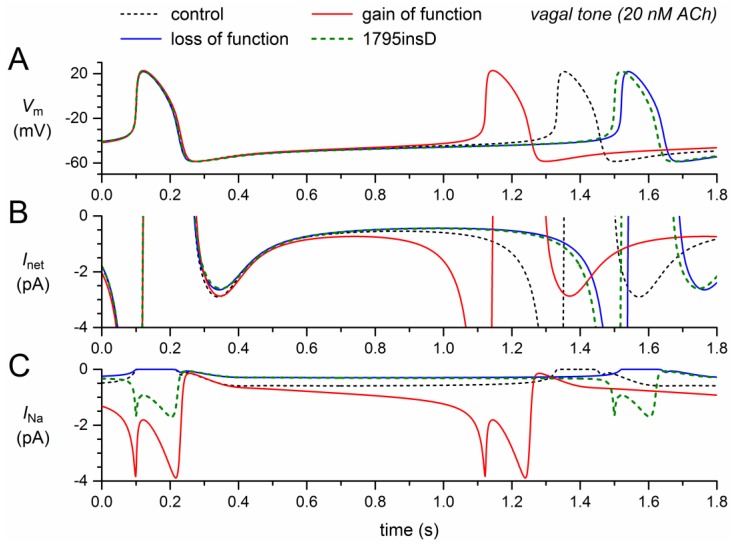
Changes in the electrical activity of the Fabbri–Severi model cell at low beating rate (vagal tone) due to loss-of-function (reduced *g_Na_* and shifts in the steady-state activation and inactivation curves) and gain-of-function (*I_pst_*) effects as observed for the 1795insD mutation in *SCN5A*. (**A**) Membrane potential (*V_m_*). (**B**) Net membrane current (*I_net_*). (**C**) Fast sodium current (*I_Na_*). Effect of the loss-of-function effects per se (blue traces), the gain-of-function effects per se (red traces), and the combined effect (dashed green traces). In the model, *I_Na_* is split into a wild-type and a mutant component, thus representing the effect of a heterozygous mutation.

The simulation results under vagal tone, as shown in Figure 5, are qualitatively, i.e., mechanistically, similar to those under normal autonomic tone. There are, however, quantitative differences. The loss-of-function effects of the mutation per se (Figure 5, blue traces) now result in a 188-ms increase in cycle length, whereas the gain-of-function effect of the mutation per se (Figure 5, red traces) now results in a 209-ms decrease in cycle length. The net effect is again a clear prolongation of the cycle length, now by 168 ms (+14%), which is considerably larger than the 6.5% prolongation under normal autonomic tone.

We also carried out simulations under β-adrenergic tone. The default beating rate of the model cell was increased from 74 to 140 beats/min through the simulated administration of isoprenaline, which, like ACh, affects several membrane ionic currents as well as the Ca^2+^ uptake by the sarcoplasmic reticulum, as detailed in Section 4.3. The simulation results were qualitatively similar to those under normal autonomic tone and vagal tone. The net effect of the 1795insD mutation was a prolongation of the cycle length by 21 ms (+4.9%).

### 2.6. Sinus Bradycardia

**Figure 6:**
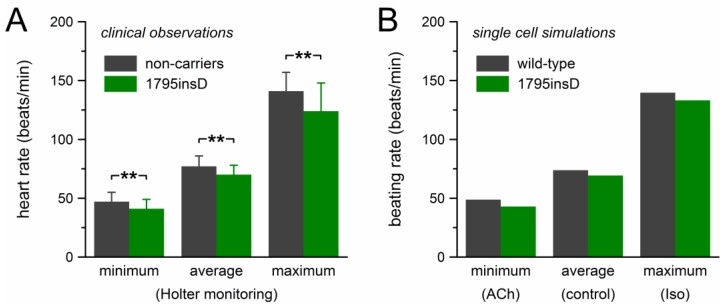
Bradycardic effect of the heterozygous 1795insD mutation. (**A**) Clinically observed heart rate of 1795insD mutation carriers [21]. Data are mean ± SD (cf. Table 1). ** *p* < 0.001. (**B**) Beating rate of the Fabbri–Severi model cell.

The mutation effects on beating rate observed in our computer simulations are summarized in Figure 6, together with the clinical observations on the heart rate of heterozygous 1795insD mutation carriers by Van den Berg et al. [21]. Figure 6A shows the clinical data of Table 1, which were obtained through 24-h Holter recordings from 54 mutation carriers and 40 non-affected family members. Bradycardic effects of the mutation were observed at all heart rates. The decrease in heart rate amounted to ≈11% and the largest effect was observed at low heart rate. The beating rate of the model cell showed a somewhat similar pattern (Figure 6B). The 12% decrease in beating rate at low heart rate closely matched the clinically observed decrease in heart rate. However, with a decrease in beating rate of 6.1% and 4.7%, respectively, the bradycardic effect at average and high rate was clearly smaller than observed clinically.

## 3. Discussion

### 3.1. Applicability of the Simulation Results

Our simulation results demonstrate how the different loss-of-function and gain-of-function effects of the 1795insD mutation in *SCN5A* compete and underlie the sinus bradycardia that is consistently observed in carriers of the mutation. However, we have to keep in mind that our simulation data are at the single-cell level, whereas the clinical data are at the whole-heart level. In our computer simulations, the complex structure of the SA node is not taken into account, neither is the electrotonic influence of the surrounding atrial tissue and the dependence thereof on the mutation of interest, which makes the atrial cells less excitable, nor are the potential pacemaker shifts upon vagal or β-adrenergic stimulation [34,35]. The complexity of the SA node has been acknowledged over the years [36,37,38,39]. Very recent studies have provided further insight into the highly specialized microanatomy of the human SA node [40,41]. These important studies may provide a framework for future 3D simulation studies on the mechanisms underlying the robust human SA nodal pacemaker function and the effects of specific mutations like 1795insD.

In our model human SA nodal cell, the loss-of-function effects of the 1795insD mutation prevail. This does not imply that the same holds for ventricular cardiomyocytes. In these cells, the sodium current is much larger than in SA nodal cells, so that the associated persistent current that flows during the plateau phase of the action potential can cause a substantial increase in action potential duration, as observed in LQT3 patients in general and carriers of the 1795insD mutation in particular, despite the mutation-induced decrease in *g_Na_*. At the same time, this mutation-induced decrease in *g_Na_* ensures that the sodium current during the upstroke of the action potential is substantially smaller than under control conditions, which can in turn explain the slowed conduction and Brugada syndrome phenotype [18].

Our simulation results are not limited to the 1795insD mutation. They are also applicable to phenotypically similar mutations in *SCN5A*, like ΔKPQ, ΔK1500 and E1784K, which show comparable loss-of-function and gain-of-function effects, i.e., a decrease in peak inward current, a persistent current, a positive shift in steady-state activation, and a negative shift in steady-state inactivation [42]. Furthermore, they can help explain the sinus bradycardia that is observed in other mutations in *SCN5A* that exhibit some of the effects of the 1795insD mutation, like the E161K mutation, which is associated with an almost threefold reduction in peak sodium current and a +11.9 mV shift in steady-state activation, but does not show a persistent current or changes in inactivation properties [43].

### 3.2. Parameter Settings

We based the parameter settings of our mutant *I_Na_* on the data from voltage clamp experiments by Bezzina et al. [18] and Veldkamp et al. [27,28]. These experiments were carried out on wild-type and 1795insD mutant channels expressed in *Xenopus* oocytes and HEK-293 cells, respectively. In either case, the electrophysiological experiments were performed at room temperature (21 °C). We cannot rule out that recordings at a more physiological temperature would have revealed additional effects of the 1795insD mutation, as has been the case for several other mutations in *SCN5A* expressed in mammalian cell lines, like T1620M, Y1795C, and Y1795H [44,45]. Recordings from Y1795C and Y1795H channels at 32 °C by Rivolta et al. [44] revealed a prominent intermediate inactivated state that was almost absent at room temperature. Similarly, Dumaine et al. [45] had primarily observed several arrhythmogenic properties of the T1620M mutation at 32 °C, like a positive shift in the steady-state activation curve and a slower recovery from inactivation. Unfortunately, data on 1795insD channel kinetics at more physiological temperatures are not available.

HEK-293 cells are often considered more reliable because of their mammalian origin, but in a comparative study on nine different sick sinus syndrome-related mutations in *SCN5A*, not including 1795insD, Gui et al. [46] found that most of the electrophysiological parameters obtained with oocytes were not different from those obtained with HEK-293 cells (all recordings made at a room temperature of 20–22 °C). However, apart from the difference in expression system (mammalian versus non-mammalian), there was also a difference in the cotransfection with β-subunits. Bezzina et al. [18] only expressed the α-subunit of the channel, whereas Veldkamp et al. [27,28] coexpressed the human Na^+^ channel β_1_-subunit. Wei et al. [47] showed that coexpression with the human Na^+^ channel β_1_-subunit may cause shifts in the voltage dependence of steady-state inactivation. It is therefore conceivable that this coexpression, rather than the difference in expression system, may play a role in the three, or four, discrepancies in experimental observations that will now be discussed.

First, the data of Bezzina et al. [18] and Veldkamp et al. [27] regarding mutation-induced changes in the time course of inactivation and recovery from inactivation show differences in several respects. However, the observed changes are relatively small [18] and reduce *I_Na_* channel availability primarily at rapid heart rates [27]. Moderate changes in inactivation kinetics will not have a large impact on the outcome of our simulations because these kinetics are fast compared to the changes in membrane potential over the time course of a human SA nodal action potential. Therefore we refrained from incorporating any changes in inactivation kinetics into our model. This may underlie the relatively small bradycardic effects at high rates (Figure 6B, rightmost bars, versus Figure 6A, rightmost bars), where mutation-induced changes in inactivation kinetics may come into play [27].

Second, Bezzina et al. [18] found a shift in steady-state activation (by +8.1 mV) as well as steady-state inactivation (by −7.3 mV), whereas Veldkamp et al. [27] only found a shift in steady-state inactivation (by −9.7 mV). We adopted the data of Bezzina et al. [18]. However, if we had adopted those of Veldkamp et al. [27], our simulation results would not have been very different. On the one hand, the absence of a positive shift in steady-state activation would result in a larger *I_pst_*, but on the other hand, it would also result in a smaller *g_Na_* which is then required to match the voltage clamp data (the smaller the shift in activation, the larger the peak current in voltage clamp), opposing the potential increase in *I_pst_*.

Third, Bezzina et al. [18] observed a striking difference in peak amplitude between their wild-type and 1795insD mutant Na^+^ current, which we took into account in our voltage clamp simulations to determine *g_Na_*, whereas Veldkamp et al. [28] observed wild-type and mutant peak currents of similar amplitude. The latter data may, however, have been biased by limits put on the peak current to improve the voltage clamp conditions. In their experiments on heterozygous *Scn5a*^1798insD/+^ mice, i.e., transgenic mice carrying the mouse equivalent (1798insD) of the human *SCN5A*-1795insD mutation, Remme et al. [48,49] found a 39% reduction in peak sodium current, which also points to a significant mutation-induced reduction in *g_Na_*. Unfortunately, as noted by Remme et al. [48], the heterozygous mice are not suitable to determine the characteristics of the mutant channels due to the prevailing wild-type channels. In particular, relatively small changes in activation and inactivation kinetics of the mutant channels, as measured in transfection systems [18,28], will be masked by these prevailing wild-type channels, explaining why Remme et al. [48] did not observe such changes. The same holds for the heterozygous *Scn5a*^1798insD/+^ mouse embryonic stem cells and induced pluripotent stem cells of Davis et al. [50], which also show a substantial reduction in peak sodium current. Homozygous mutant mice are not viable [48]. Otherwise, these could be used to directly assess the electrophysiological characteristics of the mutant channels in their native environment. Of note, the Na_V_1.5 α-subunit of the channel does not only interact with the β_1_-subunit, but also with other β-subunits and various regulatory proteins [14,51,52,53].

The observation of a persistent current by Veldkamp et al. [27,28], but not by Bezzina et al. [18], may be regarded as a fourth discrepancy. However, Bezzina et al. [18] already “considered the possibility that a persistent inward current, usually <2% of the peak current, was too small to distinguish,” thus pointing to the technical limitations that hampered the discovery of a relatively small persistent current in their experiments.

### 3.3. Previous Simulation Studies

Our simulations are certainly not the first to assess the effects of a specific mutation in *SCN5A* on the electrical activity of cardiac myocytes. However, in most studies conducted thus far, e.g., [54,55,56,57,58,59,60,61,62,63,64,65,66,67,68,69,70,71], the emphasis was on ventricular cells, some also considering atrial or Purkinje cells. Fewer studies, e.g., by Butters et al. [72], Wu et al. [73], and Zhang et al. [74], focused on the effects on SA nodal cells, using the rabbit SA nodal cell models by Zhang et al. [75,76]. In our original study of the effects of the 1795insD mutation on SA nodal cells [28], we also used a rabbit SA nodal cell model [77]. These rabbit models, in particular those of Zhang et al. [75,76], do not only have a considerably shorter cycle length than the human model used in the present study, but also a different action potential configuration with a much shorter diastolic phase. As a result, the role of gain-of-function effects of a mutation, which prolong the action potential duration, may have been exaggerated in previous simulation studies, whereas the role of loss-of-function effects, which act on the diastolic phase, may have been underestimated. This also holds for our own study of the effects of the 1795insD mutation [28] and underscores the importance of the availability of a human SA nodal pacemaker cell model with an action potential configuration that closely resembles that of isolated human SA nodal pacemaker cells.

The recent development of a comprehensive model of a human SA nodal cell by Fabbri et al. [33] allowed us to assess the effects of the 1795insD mutation in a more realistic action potential configuration, with a much longer diastolic depolarization phase, than we could use before. In our original study [28], we did not take into account that the 1795insD mutation strongly reduces *g_Na_*, as became irrefutable by the studies on transgenic mice by Remme et al. [48,49]. Furthermore, *I_pst_* was overestimated because it was modelled as a fraction of 1–3% non-inactivating channels, whereas a fraction of 0.6% can be sufficient to arrive at a persistent current with an amplitude of 1.4% of the peak current, as shown in Section 2.1. Therefore, a direct comparison with our present study is not possible. We can, however, conclude that the originally observed decrease in sinus rate through an increase in action potential duration [28] is much less important than anticipated, also because of the much longer diastolic phase of human SA nodal cells compared to rabbit SA nodal cells, which reduces the effect of an increase in action potential duration on rate.

The outcome of our simulations is, of course, dependent on the implementation of *I_Na_*, as well as other individual ion currents, in the Fabbri–Severi model. Also, we have to keep in mind that there is no hyperpolarizing effect of the surrounding atrial tissue in our single cell simulations. Such hyperpolarizing effect may affect the interaction between the individual ion currents, e.g., by increasing the availability of (mutant) sodium channels. Yet, the Fabbri–Severi model appears a useful tool to gain insight into the complex interaction between loss-of-function and gain-of-function effects of a specific mutation in *SCN5A*. 

### 3.4. Future Directions

It seems challenging to repeat the experiments by Bezzina et al. [18] and Veldkamp et al. [27,28] under close-to-physiological conditions to unambiguously determine the effects of the 1795insD mutation. Also, action potential clamp experiments with the use of the action potentials recorded from isolated humans SA node cells by Verkerk et al. [78] may be helpful to further understand how wild-type and mutant sodium currents behave during a human SA nodal action potential. Another option is the use of dynamic clamp experiments in which the Fabbri–Severi model cell can interact in real time with cells expressing wild-type or 1795insD mutant Na_V_1.5 channels.

## 4. Materials and Methods

### 4.1. Computational Model of a Single Human SA Nodal Pacemaker Cell

The spontaneous electrical activity of a single human SA nodal pacemaker cell was simulated using the comprehensive mathematical model of such cell that was recently developed by Fabbri et al. [33]. This model contains Hodgkin and Huxley-type equations for the fast sodium current. These read:

(1) $$I_{Na}=m^{3}\cdot h\cdot g_{Na}\cdot \left(V_{m}-E_{mh}\right),$$

(2) $$E_{mh}=\;\frac{R\cdot T}{F}\;\cdot \ln\frac{{\left[{\mathrm{Na}}^{+}\right]}_{e}+0.12\cdot {\left[K^{+}\right]}_{e}\;}{{\left[{\mathrm{Na}}^{+}\right]}_{i}+0.12\cdot {\left[K^{+}\right]}_{i}},$$

(3) $$m_{\infty }=\;\frac{1}{1+e^{\frac{-\left(V_{m}+42.0504\right)}{8.3106}}},$$

(4) $$\alpha_{m}=200\cdot \;\frac{V_{m}+41}{1-e^{-0.1\cdot \left(V_{m}+41\right)}},$$

(5) $$\beta_{m}=8000\cdot e^{-0.056\cdot \left(V_{m}+66\right)},$$

(6) $$\tau_{m}=\frac{1}{\alpha_{m}+\beta_{m}},$$

(7) $$\frac{dm}{dt}=\;\frac{m_{\infty }-m}{\tau_{m}},$$

(8) $$h_{\infty }=\;\frac{1}{1+e^{\frac{V_{m}+69.804}{4.4565}}},$$

(9) $$\alpha_{h}=20\cdot e^{-0.125\cdot \left(V_{m}+75\right)},$$

(10) $$\beta_{h}=\frac{2000}{1+320\cdot e^{-0.1\cdot \left(V_{m}+75\right)}},$$

(11) $$\tau_{h}=\frac{1}{\alpha_{h}+\beta_{h}},$$

(12) $$\frac{dh}{dt}=\;\frac{h_{\infty }-h}{\tau_{h}}\;.$$

In Equation (1), the membrane potential (*V_m_*) and reversal potential (*E_mh_*) are in mV, whereas the sodium current (*I_Na_*) and fully-activated conductance (*g_Na_*) are in pA and nS, respectively; *m* and *h* are the activation and inactivation gating variables, respectively. The fully-activated conductance *g_Na_* amounts to 22.3 nS. As can be inferred from Equation (1), *I_Na_* does not have a persistent component under control conditions.

The reversal potential of *I_Na_* (i.e., *E_mh_*) is computed with the universal gas constant *R* of 8.314 J∙mol^−1^∙K^−1^, the Faraday constant *F* of 96.485 C·mmol^−1^, the absolute temperature *T* of 310 K, the extracellular sodium concentration [Na^+^]_e_ of 140 mmol·L^−1^, the extracellular potassium concentration [K^+^]_e_ of 5.4 mmol·L^−1^, the intracellular sodium concentration [Na^+^]_i_ of 5.0 mmol·L^−1^, and the intracellular potassium concentration [K^+^]_i_ of 140 mmol·L^−1^, and amounts to 49.8 mV (Equation (2)). Equations (3)–(7) describe the time and voltage dependence of the activation gating variable *m*. Its steady-state value *m_∞_* is given by Equation (3), whereas Equations (4)–(7) show the rate constants *α_m_* and *β_m_* (both expressed in s^−1^), the associated time constant *τ_m_* (in s), and the first-order differential equation for *m*. Similarly, Equations (8)–(12) describe the time and voltage dependence of the inactivation gating variable *h*.

### 4.2. Implementation of the 1795insD Mutation

Effects of the 1795insD mutation in *SCN5A* were implemented in the CellML code [79] of the Fabbri–Severi model. To this end, the model *I_Na_* was split into a wild-type and a mutant component (*I_Na,WT_* and *I_Na,mut_*, respectively), thus representing the heterozygous nature of the mutation. Accordingly, *I_Na_*_,WT_ was computed through Equations (1)–(12), but with *g_Na_* reduced to 50% of its control value, i.e., with a *g_Na_* value of 11.15 nS instead of 22.3 nS.

The mutant component of *I_Na_* (i.e., *I_Na,mut_*) differed from the wild-type component in several respects. First, the voltage dependence of the equations governing the *I_Na_* activation and inactivation kinetics was shifted by +8.1 and −7.3 mV, respectively, to implement the experimentally observed +8.1 and −7.3 mV shifts in the steady-state activation and inactivation curves [18]. Next, the fully-activated conductance (*g_Na,mut_*) was scaled by 50%, i.e., to 25% of the control value, to implement the experimentally observed reduction in peak current [18], as set out in the Results section. Furthermore, 0.6% of the mutant *I_Na_* channels were made non-inactivating to introduce a persistent component, i.e., *I_pst_*, with an amplitude of 1.0–1.5% of the peak current recorded in voltage clamp mode, in accordance with the experimentally observed *I_pst_* [27,28]. The resulting equations for *I_Na,mut_*, as far as they differ from Equations (1)–(12), read:

(13) $$I_{Na,mut}=m^{3}\cdot h\cdot \left(1-f\right)\cdot g_{Na,mut}\cdot \left(V_{m}\;-\;E_{mh}\right)+m^{3}\cdot f\cdot g_{Na,mut}\cdot \left(V_{m}\;-\;E_{mh}\right),$$

(14) $$I_{pst}=m^{3}\cdot f\cdot g_{Na,mut}\cdot \left(V_{m}\;-\;E_{mh}\right),$$

(15) $$m_{\infty }=\;\frac{1}{1+e^{\frac{-\left((V_{m}-8.1\right)+42.0504)}{8.3106}}},$$

(16) $$\alpha_{m}=200\cdot \;\frac{\left(V_{m}-8.1\right)+41}{1-e^{-0.1\cdot \left(\left(V_{m}-8.1\right)+41\right)}},$$

(17) $$\beta_{m}=8000\cdot e^{-0.056\cdot (\left(V_{m}-8.1)+66\right)},$$

(18) $$h_{\infty }=\;\frac{1}{1+e^{\frac{(V_{m}+7.3)+69.804}{4.4565}}},$$

(19) $$\alpha_{h}=20\cdot e^{-0.125\cdot (\left(V_{m}+7.3)+75\right)},$$

(20) $$\beta_{h}=\frac{2000}{1+320\cdot e^{-0.1\cdot \left(\left(V_{m}+7.3\right)+75\right)}}.$$

with the fully-activated conductance *g_Na,mut_* and the fraction of non-inactivating channels (*f*) of Equations (13) and (14) set to 5.575 nS and 0.6%, respectively. A small fraction of the mutant channels is made non-inactivating by fixing their gating variable *h* to 1, thus creating a non-zero persistent component of *I_Na,mut_*, i.e., *I_pst_*, whereas the remaining channels (fraction 1 − *f*) do inactivate (Equations (13) and (14)). The voltage dependence of *m*_∞_, α*_m_*, and *β_m_* is shifted by +8.1 mV (Equations (15)–(17)) and that of *h*_∞_, α*_h_*, and *β_h_* by −7.3 mV (Equations (18)–(20)). The resulting shift in voltage dependence of *τ_m_* and *τ_h_* had a negligible effect on the outcome of the simulations; the main effect of the mutation is through its effect on *m*_∞_ and *h*_∞_. No attempts were made to implement the experimentally observed changes in inactivation kinetics [18,27,28].

### 4.3. Implementation of Vagal and β-Adrenergic Tone

The default Fabbri–Severi model has a beating rate of 74 beats/min, which can be varied through the simulated administration of acetylcholine (*ACh*) or isoprenaline (Iso), reflecting the autonomic modulation of pacemaking [33]. The control beating rate of 74 beats/min was lowered to 49 beats/min (vagal tone) through the simulated administration of 20 nmol/L *ACh*. A beating rate of 140 beats/min (β-adrenergic tone) was obtained through the simulated administration of Iso, tuning the model parameters affected by Iso to arrive at this beating rate.

In the Fabbri–Severi model, ACh acts through activation of the ACh-activated K^+^ current (*I_K,ACh_*) as well as inhibition of the hyperpolarization-activated ‘funny current’ (*I*_f_), the L-type calcium current (*I_Ca,L_*), and the sarco/endoplasmic reticulum Ca^2^^+^-ATPase (SERCA pump). Under control conditions, *I_K,ACh_* is zero, but in the presence of ACh it is computed through:

(21) $$I_{K,ACh}=a\cdot g_{K,ACh}\cdot \left(V_{m}-E_{K}\right)\cdot \left(1+e^{\frac{V_{m}+20}{20}}\right),$$

(22) $$E_{K}=\;\frac{R\cdot T}{F}\;\cdot \ln\frac{{\left[K^{+}\right]}_{e}}{{\left[K^{+}\right]}_{i}},$$

in which the maximal conductance *g_K,ACh_* and reversal potential *E_K_* amount to 3.44 nS and −87.0 mV, respectively. The time and voltage dependence (and ACh dependence) of the gating variable *a* is described by:

(23) $$\alpha_{a}=0.025641+\;\frac{3.573159}{1+\frac{18003.42}{{\left[ACh\right]}^{1.6951}}},$$

(24) $$\beta_{a}=10\cdot e^{0.0133\cdot \left(V_{m}+40\right)},$$

(25) $$a_{\infty }=\frac{\alpha_{a}}{\alpha_{a}+\beta_{a}},$$

(26) $$\tau_{a}=\frac{1}{\alpha_{a}+\beta_{a}},$$

(27) $$\frac{da}{dt}=\;\frac{a_{\infty }-a}{\tau_{a}},$$

with the rate constants α*_a_* and *β_a_* both expressed in s^−1^, the associated time constant *τ_a_* in s, and the ACh concentration [*ACh*] in nmol/L. 

ACh affects *I*_f_ by shifting the voltage dependence of its steady-state activation curve and its time constant in the negative direction along the voltage axis. This shift (*ACh_shift_*) is computed through

(28) $$ACh_{shift}=-1\;-\;9.898\;\cdot \;\frac{{\left[ACh\right]}^{0.618}}{{\left[ACh\right]}^{0.618}+\;6.24976},$$

in which *ACh_shift_* is expressed in mV and [*ACh*] in nmol/L. The shift amounts to −6.0 mV at the simulated [ACh] of 20 nmol/L, thus considerably inhibiting *I*_f_. *I*_Ca,L_ is also inhibited by ACh. The amount of block is expressed as a fraction *ACh_block_* that is computed through:

(29) $$ACh_{block}=0.31\;\cdot \;\frac{\left[ACh\right]}{\left[ACh\right]+\;90},$$

in which [*ACh*] is again expressed in nmol/L. *ACh_block_* amounts to 5.6% at the simulated [*ACh*] of 20 nmol/L. A similar equation holds for the amount of block of the SERCA pump, *b_up_*:

(30) $$b_{up}=0.7\;\cdot \;\frac{\left[ACh\right]}{\left[ACh\right]+\;90},$$

which amounts to 12.7% at the simulated [*ACh*] of 20 nmol/L.

In the Fabbri–Severi model, Iso acts through increasing effects on *I_f_*, *I_Ca,L_*, and the SERCA pump, as opposed to ACh. Furthermore, Iso increases the sodium-potassium pump current (*I_NaK_*) and the slow delayed rectifier potassium current (*I_Ks_*). The associated parameters are tuned, all to the same extent, to arrive at a specific beating rate, as set out by Fabbri et al. [33]. In our case, the parameters were tuned to obtain a rate of 140 beats/min. The voltage dependence of the steady-state activation and time constant of *I*_f_ was shifted along the voltage axis by +10 mV. The maximal conductance of *I_Ca,L_* was increased by 64%. Furthermore, its steady-state activation curve was changed in two respects. It was shifted along the voltage axis by −10.67 mV and its slope factor was reduced by 29.244%. The maximal activity of the SERCA pump was increased by 25% and that of *I_NaK_* by 60%. The fully-activated conductance of *I_Ks_* was also increased by 60%. Furthermore, the steady-state activation and time constant curves of *I_Ks_* were shifted by −18.7 mV along the voltage axis, thus further increasing *I_Ks_*. Altogether, these increasing effects on *I_f_*, *I_Ca,L_*, *I_Ks_*, *I_NaK_*, and the SERCA pump resulted in an increase in beating rate to 140 beats/min.

### 4.4. Computer Simulations

The CellML code of the Fabbri–Severi model, as available from the CellML Model Repository [80,81], was edited and run in version 0.9.31.1409 of the Windows based Cellular Open Resource (COR) environment [82]. All simulations were run for a period of 100 s, which appeared a sufficiently long time to reach steady-state behaviour. The analyzed data are from the first complete action potentials after a simulated period of 90 s.

## 5. Conclusions

We conclude that the experimentally identified mutation-induced changes in *I_Na_* can explain the clinically observed sinus bradycardia in carriers of the 1795insD mutation in the *SCN5A* gene. Furthermore, we conclude that the Fabbri–Severi model may prove a useful tool in understanding cardiac pacemaker activity in humans.

## Figures and Tables

**Table 1:** Heart rate in carriers of the 1795insD mutation in the *SCN5A* gene.

Group	Heart Rate in 24-h Holter Recordings (beats/min)			*n*
	Minimum	Average	Maximum	
Mutation carriers	41 ± 8 **	70 ± 8 **	124 ± 24 **	54
Non-carriers	47 ± 8	77 ± 9	141 ± 16	40

Data are mean ± SD [21]. ** *p* < 0.001 vs. non-affected family members (“non-carriers”).

**Table 2:** Effects on action potential configuration.

Condition	CL (ms)	∆CL (ms)	APD_90_ (ms)	∆APD_90_ (ms)
Control	813.4		151.0	
Activation shift	859.8	+46	150.7	−0.2
Inactivation shift	857.0	+44	150.7	−0.2
Double shift	866.3	+53	150.7	−0.3
Reduced *g_Na_*	839.8	+26	150.8	−0.2
*I_pst_*	747.4	−66	157.4	+6.5
*I_pst_* & reduced *g_Na_*	800.9	−12	154.0	+3.1
Loss of function	867.1	+54	150.6	−0.3
Gain of function	747.4	−66	157.4	+6.5
1795insD	866.3	+53	153.0	+2.0

CL: cycle length; APD_90_: action potential duration at 90% repolarization.

## Abbreviations

*ACh*	Acetyl-choline
*g_Na_*	Conductance of *I_Na_*
*I_Na_*	Fast sodium current
*I_net_*	Net membrane current
*I_pst_*	Persistent, non-inactivating component of *I_Na_*
Iso	Isoprenaline
LQT3	Long-QT syndrome type 3
LQTS	Long QT syndrome
Na_V_1.5	Pore-forming α-subunit of the cardiac voltage-gated sodium channel
*SCN5A*	Gene encoding the Na_V_1.5 protein
*V_m_*	Membrane potential
